# Genome-wide Two-marker linkage disequilibrium mapping of quantitative trait loci

**DOI:** 10.1186/1471-2156-15-20

**Published:** 2014-02-08

**Authors:** Jie Yang, Wei Zhu, Jiansong Chen, Qiao Zhang, Song Wu

**Affiliations:** 1 Department of Preventive Medicine, Stony Brook University, Stony Brook, NY 11790, USA; 2 Department of Applied Mathematics and Statistics, Stony Brook University, Stony Brook, NY 11790, USA

**Keywords:** Genetic mapping, Linkage disequilibrium mapping, Linked loci, Genome wide association study

## Abstract

**Background:**

In a natural population, the alleles of multiple tightly linked loci on the same chromosome co-segregate and are passed non-randomly from generation to generation. Capitalizing on this phenomenon, a group of mapping methods, commonly referred to as the linkage disequilibrium-based mapping (LD mapping), have been developed recently for detecting genetic associations. However, most current LD mapping methods mainly employed single-marker analysis, overlooking the rich information contained within adjacent linked loci.

**Results:**

We extend the single-marker LD mapping to include two linked loci and explicitly incorporate their LD information into genetic mapping models (tmLD). We establish the theoretical foundations for the tmLD mapping method and also provide a thorough examination of its statistical properties. Our simulation studies demonstrate that the tmLD mapping method significantly improves the detection power of association compared to the single-marker based and also haplotype based mapping methods. The practical usage and properties of the tmLD mapping method were further elucidated through the analysis of a large-scale dental caries GWAS data set. It shows that the tmLD mapping method can identify significant SNPs that are missed by the traditional single-marker association analysis and haplotype based mapping method. An R package for our proposed method has been developed and is freely available.

**Conclusions:**

The proposed tmLD mapping method is more powerful than single marker mapping generally used in GWAS data analysis. We recommend the usage of this improved method over the traditional single marker association analysis.

## Background

Most economically, biologically and clinically important traits, such as those linked to poplar growth, cancer development and dental caries risk, are inherently complex in terms of their polygenic control and sensitivity to the environment [1]. The number of genes involved in these traits is typically large, each exerting a small effect and acting singly or interactively with others in a complicated network. For this reason, the genetic analysis of complex traits has been very difficult. However, a profound understanding of the genetic control mechanisms of complex traits is crucial to economy and life. Therefore, the development of more powerful and complex genetic mapping methods has become increasingly urgent.

In recent years, with the advancement of new DNA-based biotechnologies, such as single-nucleotide polymorphism (SNP) arrays, genome-wide association studies (GWAS) have become feasible to dissect the phenotypic variation of a complex trait into individual genetic components. Particularly, SNP arrays have gained popularity due to their cost-effectiveness: in year 2011 alone, 1068 GWAS were performed, each with at least 100,000 SNPs genotyped (http://www.genome.gov/gwastudies). Based on the most recent summary data of dbSNP database (http://www.ncbi.nlm.nih.gov/projects/SNP), there are ~ $38 million (about 1 percent of the total genome) of validated SNPs in human genome. However, even the densest SNP array on the market can only accommodate ~1 million SNPs, and hence a great percentage of SNPs is not able to be sampled in a real genetic study. Fortunately, SNPs in the genome are not independent from each other, *i.e.* they are locally connected and form the so-called linkage disequilibrium (LD) blocks. Because of this unique correlation structure, the sampled genetic markers carry partial information about the unsampled SNPs and may be used for genomewide association analyses.

LD is a phenomenon arising from the co-inheritance of alleles at nearby loci on the same chromosome, and is defined as the deviation of the observed frequency of a haplotype from random association [2]. Historically, LD analysis was developed to quantify the genetic structure and the diversity of natural populations [3-5]. Many efforts have been put into developing dense maps of molecular markers for a wide variety of species. For example, LD structures have been estimated in human [6] as well as Holstein cattle [7], sheep [8] and dog [9]. With some regularity conditions [2], it can be shown that a LD value between any two loci decays with generations at the recombination rate between them:

(1) $$D^{\left(t+1\right)}=\left(1-r\right)D^{\left(t\right)}$$

where *D*^(*t*+1)^ is the LD value at generation *t* + 1 and *r* is the recombination rate between the two loci. Therefore, the LD value approaches to zero gradually at a geometric rate of 1-*r*. The larger the *r*, the faster the rate of convergence. According to Equation ([1]), if a significant *D*^(*t*+1)^ value can be detected in the current generation, it implies *r* must be very small, almost close to 0, under the assumption that the initial LD was generated long time ago (*i.e. t* is large). This assumption is plausible because it does take a long time for mutations/LD to be spread in a population. Therefore, the principle of linkage disequilibrium decaying with generation builds up an alternative mapping strategy [10,11], which provides an important tool for the fine mapping of genes affecting a quantitative trait.

The LD mapping based on a single marker has been greatly studied [12-14]. However, little effort has been put on the LD mapping with multiple markers. Motivated by the seminal work of interval mapping proposed by Lander and Botstein in 1989 [15], in which genetic mapping was performed based on two neighboring genetic markers in controlled experiments, we propose to develop a new LD mapping framework that utilizes two SNP markers in a natural population. The new model explicitly incorporates the LD information between two markers into the mapping analysis, and thus we expect the analysis based on two markers is more powerful than that based on a single marker in a natural population just as Lander and Botstein have discovered in the controlled experiment. In the following sections, we first laid out the modeling framework for the two-marker LD mapping (tmLD), with details on parameter estimation and hypothesis testing. We then further elucidated our method through extensive simulation studies. Finally, we applied our method to a GWAS dental caries data set, followed by some discussions.

## Methods

### Two-marker LD (tmLD) mapping

In the tmLD mapping framework, we assume a dichotomous quantitative trait locus (QTL, ) of alleles *Q* and *q* that is causal but unobserved, and the allele frequencies of *Q* and *q* are expressed as *p*_2_ and 1-*p*_2_. Suppose that this QTL is genetically associated with two genotyped SNP markers, ℳ_1_ and ℳ_2__,_ of two alleles *M*_1_ and *m*_1_, and *M*_2_ and *m*_2_, with corresponding frequencies of *p*_1_ and 1-*p*_1_, and *p*_3_ and 1-*p*_3_, respectively. Further suppose the three linked SNPs in a tandem order, ℳ_1_,  and ℳ_2_ at loci 1, 2 and 3, and the recombination rates between ℳ_1_ and , between  and ℳ_2_, and between ℳ_1_ and ℳ_2_ are *r*_12_, *r*_23_ and *r*_13_, respectively. The three SNPs form 8 possible haplotypes:  *M*_1_*QM*_2_ (111), *M*_1_*Qm*_2_ (110), *M*_1_*qM*_2_ (101), *M*_1_*qm*_2_ (100), *m*_1_*QM*_2_ (011), *m*_1_*Qm*_2_ (010), *m*_1_*qM*_2_ (001), *m*_1_*qm*_2_ (000). To describe the linkage disequilibrium among them, their frequencies can be represented as follows using four trigenic disequilibria parameters *D*_12_, *D*_23_, *D*_13_ and *D*_123_ (Additional file 1):

(2) $$p_{\mathrm{ijk}}=p_{1}^{i}{\left(1-p_{1}\right)}^{1-i}p_{2}^{j}{\left(1-p_{2}\right)}^{1-j}p_{3}^{k}{\left(1-p_{3}\right)}^{1-k}+D_{\mathrm{ijk}}$$

and $D_{\mathrm{ijk}}=\frac{1}{2}\left[{\left(-1\right)}^{\left|i-j\right|}D_{12}+{\left(-1\right)}^{\left|j-k\right|}D_{23}+{\left(-1\right)}^{\left|i-k\right|}D_{13}-{\left(-1\right)}^{\left|i+j+k-1\right|}D_{123}\right]$ where *i*, *j*, *k* = 0, 1, *D*_12_, *D*_23_, *D*_13_ have exactly the same meaning as those in digenic disequilibria models for loci at positions 1/2, 2/3 and 1/3; and *D*_123_ is an additional trigenic disequilibria parameter for three loci together. Model (1) implies that *D*_12_, *D*_23_, *D*_13_ all geometrically decay with generations. It can be shown that with some reasonable assumptions, the *D*_123_ decreases with generations at a rate of (1-*r*_13_) and therefore also changes very slowly with time (Additional file 2). Hence, significant *D*_12_, *D*_23_,  and *D*_123_ at current generation imply  *r*_12_and *r*_23_ are very small, which form the basis for LD mapping using two genetic markers.

### Likelihood function

Suppose there is a random sample of size *n* drawn from a natural human population at Hardy–Weinberg equilibrium. In this sample, multiple polymorphic sites, e.g. single nucleotide polymorphism (SNP), are genotyped, aiming at the identification of QTL affecting a continuous trait. The relationship between the observed phenotypic values and their expected means, determined by QTL genotypes, can then be described by the following model,

(3) $$y_{i}=\sum_{j=0}^{2}\xi_{\mathrm{ij}}\mu_{j}+e_{i},\;i=1,\ldots ,n\;$$

Where *y*_*i*_ is the phenotypic values for subject *i*, ξ_ij_ is an indicator variable defined as 1 if subject *i*, which contains markers (ℳ_*i*1_, ℳ_*i*2_), has a QTL genotype *j* (*j* = 2 for *QQ*, 1 for *Qq* and 0 for *qq*) and 0 otherwise, *μ*_*j*_ is the expected phenotypic value for QTL genotype *j*, and *e*_*i*_ is the error term reflecting the polygenic effects of other unlinked genes and the environmental effect, which can be assumed to follow *N*(0, *σ*^2^) if *y* is continuous. The conditional probability of subject *i* with its given markers carrying a certain QTL genotype *j*, $\;\pi_{j|i}=P\left(Q=j|{ℳ}_{i1},{ℳ}_{i2}\right)$ or *P*(*ξ*_*ij*_ = 1), can be calculated from Table 1. Therefore, the likelihood of the quantitative trait (*y*) and molecular markers (ℳ_1_, ℳ_2_) for one putative QTL  and can be constructed by a mixture model:

$$\;L\left(\Omega_{p},\Omega_{q}|y,{ℳ}_{1},{ℳ}_{2}\right)=\prod_{i=1}^{n}\sum_{j=0}^{2}\pi_{j|i}f_{j}\left(y_{i}|\Omega_{q}\right),$$

where Ω_*p*_ is a vector of the population genetic parameters (*p*_1_, *p*_2_, *p*_3_, *D*_12_, *D*_23_, *D*_13_, *D*_123_) that is used to describe frequencies of haplotypes formed by markers and QTL and subsequently *π*_*j*|*i*_ s, Ω_*q*_ is a vector of the quantitative genetic parameters that define genotype-specific traits, which contains (*μ*_*j*_, *j* = 1,  2, 3, and *σ*) for a continuous trait that is assumed to be normally distributed, and *f*_*j*_(∙) is the probability density function for QTL genotype *j*.

**Table 1:** Joint zygote probabilities of the QTL genotypes at QTL Q and two-marker genotypes at markers M1 and M2, as expressed in terms of zygote configurations in a natural population

**Marker**			**Joint marker-QTL genotype frequency**		
**Genotype**		**Frequency**	***qq*** **(0)**	***Qq*** **(1)**	***QQ*** **(2)**
*m*_1_*m*_1_*m*_2_*m*_2_	(00)	$p_{00}^{2}$	$p_{000}^{2}$	2*p*_010_*p*_000_	$p_{010}^{2}$
			(*n*_000_)	(*n*_010_)	(*n*_020_)
*m*_1_*m*_1_*M*_2_*m*_2_	(01)	2*p*_01_*p*_00_	2*p*_001_*p*_000_	2*p*_011_*p*_000_ + 2*p*_010_*p*_001_	2*p*_010_*p*_011_
			(*n*_001_)	(*n*_011_)	(*n*_021_)
*m*_1_*m*_1_*M*_2_*M*_2_	(02)	$p_{01}^{2}$	$p_{001}^{2}$	2*p*_011_*p*_001_	$p_{011}^{2}$
			(*n*_002_)	(*n*_012_)	(*n*_022_)
*M*_1_*m*_1_*m*_2_*m*_2_	(10)	2*p*_00_*p*_10_	2*p*_100_*p*_000_	2*p*_110_*p*_000_ + 2*p*_100_*p*_010_	2*p*_110_*p*_010_
			(*n*_100_)	(*n*_110_)	(*n*_120_)
*M*_1_*m*_1_*M*_2_*m*_2_	(11)	2*p*_11_*p*_00_	2*p*_101_*p*_000_ + 2*p*_100_*p*_001_	2*p*_111_*p*_000_ + 2*p*_110_*p*_001_	2*p*_111_*p*_010_ + 2*p*_110_*p*_011_
		+ 2*p*_10_*p*_01_		+ 2*p*_101_*p*_010_ + 2*p*_100_*p*_011_	
			(*n*_101_)	(*n*_111_)	(*n*_121_)
*M*_1_*m*_1_*M*_2_*M*_2_	(12)	2*p*_11_*p*_01_	2*p*_101_*p*_001_	2*p*_111_*p*_001_ + 2*p*_101_*p*_011_	2*p*_111_*p*_011_
			(*n*_102_)	(*n*_112_)	(*n*_122_)
*M*_1_*M*_1_*m*_2_*m*_2_	(20)	$p_{10}^{2}$	$p_{100}^{2}$	2*p*_110_*p*_100_	$p_{110}^{2}$
			(*n*_200_)	(*n*_210_)	(*n*_220_)
*M*_1_*M*_1_*M*_2_*m*_2_	(21)	2*p*_11_*p*_10_	2*p*_101_*p*_100_	2*p*_111_*p*_100_ + 2*p*_110_*p*_101_	2*p*_110_*p*_111_
			(*n*_201_)	(*n*_211_)	(*n*_221_)
*M*_1_*M*_1_*M*_2_*M*_2_	(22)	$p_{11}^{2}$	$p_{101}^{2}$	2*p*_111_*p*_101_	$p_{111}^{2}$
			(*n*_202_)	(*n*_212_)	(*n*_222_)

The likelihood function provides a model for obtaining the maximum likelihood estimates of the unknown parameters  (Ω_*p*_, Ω_*q*_), which can be achieved by differentiating the log-likelihood with respect to each unknown parameter, setting the derivatives equal to zero and then solving the equations. The log-likelihood function of the phenotypic values is given by

$$\ell =\log\left[L\left(\Omega_{p},\Omega_{q}|y,{ℳ}_{1},{ℳ}_{2}\right)\right]=\sum_{i=1}^{n}\log\left[\sum_{j=0}^{2}\pi_{j|i}f_{j}\left(y_{i}|\Omega_{q}\right)\right]$$

### Computational algorithms

Within the maximum likelihood estimation framework, an efficient EM algorithm can be implemented to obtain the MLEs of (Ω_*p*_, Ω_*q*_), and is summarized into the following steps:

Step 1. Give initial values for the unknown parameters (Ω_*p*_, Ω_*q*_);

Step 2. **E** step – Calculate the posterior probabilities for each subject *i* to carry a particular QTL genotype *j* using the equation $\Pi_{j|i}=\frac{\pi_{j|i}f_{j}\left(y_{i}|\Omega_{q}\right)}{\sum_{j=0}^{2}\pi_{j|i}f_{j}\left(y_{i}|\Omega_{q}\right)}.$

Step 3. M step – Solve the log-likelihood equations for each parameter based on observed data and Π_*j*|*i*_ to obtain its estimate. To estimate the quantitative genetic parameters (Ω_*q*_), their expressions in closed forms can be derived based on the estimation equations. For the estimates of the population genetic parameters (Ω_*p*_), another inner layer of EM algorithm can be employed.

Step 4. Repeat the **E** and **M** steps until the estimates converge to stable values. The estimates at convergence are the MLEs of parameters.

The detailed derivation for the EM algorithm is given in Additional file 3.

### Hypothesis testing

In general, the hypothesis testing of QTL mapping includes two steps: (1) the existence of QTL and (2) their locations. The focus of this study is on the second step, assuming that sufficient evidences for the existence of QTL have been collected to enable a large-scale genotyping study. Then the hypotheses for the tmLD method can be formulated as follows:

$$\begin{array}{c} H_{0}:\mathrm{The}\;\mathrm{QTL}\;\mathrm{is}\;\mathrm{not}\;\mathrm{associated}\;\mathrm{with}\;\mathrm{two}\;\mathrm{SNP}\;\mathrm{markers},i.e.D_{12}=D_{23}=D_{123}=0:\\ H_{1}:\mathrm{Not}\;H_{0} \end{array}$$

The estimates of the parameters under the null hypotheses can be obtained with the same EM algorithm derived for the alternative hypotheses, but with a constraint that all subjects have the same posterior probability. A likelihood ratio test (LRT) statistics can be constructed and computed to draw the inference about whether a QTL may be associated with given markers. Under the *H*_0_, the LRT statistics asymptotically follows a *χ*^2^-distribution with three degrees of freedom.

## Results

### Simulation settings

**Figure 1:**
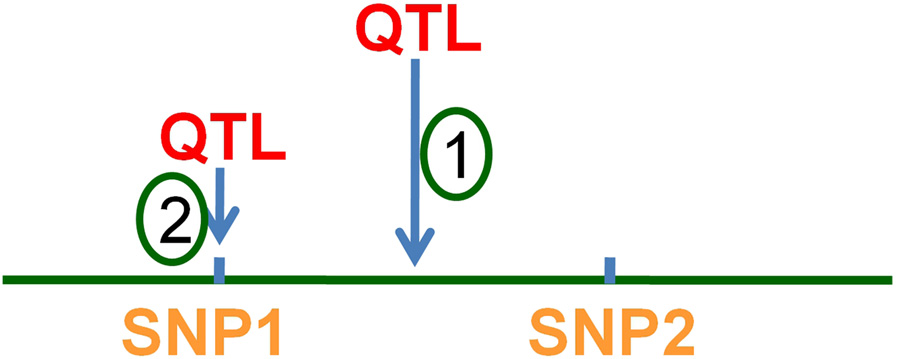
**Two simulation settings.** (1) QTL is unobserved but in linkage equilibrium with two adjacent SNPs. (2) QTL is observed as one of the SNP markers.

Extensive Monte Carlo simulation experiments were performed to examine the statistical properties of the proposed tmLD mapping method. Since in a genome-wide scan, a QTL must be located between some pair of markers, in the experimental design of simulations, we considered two scenarios as illustrated in Figure 1: (1) the QTL is assumed to be unobserved, but it is in LD with two adjacent SNPs; and (2) the QTL is assumed to be one of the genetic markers and therefore genotyped.

Let us randomly choose a sample of *n* subjects from a human population at Hardy-Weinberg equilibrium. In this population, one QTL is segregating and is inferred by a pair of markers. The allele frequencies of the markers (ℳ_1_  and  ℳ_2_) and QTL () and their linkage disequilibria values are given as follows: *p*_1_ = 0.5 for allele *M*_1_ of ℳ_1_; *p*_2_ = 0.5 for allele *Q* of ; *p*_3_ *=* 0.5 for allele *M*_2_ of ℳ_2_. The LD parameters among the markers and QTL loci are given as: *D*_12_ = 0.05, *D*_13_ = 0.15, *D*_23_ = 0.05 and  *D*_123_ = 0.04. For subjects who carry QTL genotype *j*, their phenotypic values were simulated based on Model (3), with *μ*_2_ = 10, *μ*_1_ = 5, *μ*_0_ = 0. The variances in phenotypic values were calculated based on different heritability values (*H*^2^). *H*^2^ quantifies the genetic contribution from the QTL to the overall trait and *H*^2^ = 0 implies that the means for three QTL genotype groups are the same, which are set to be 0. With the above given parameters and design, we simulated the phenotypic and marker information by assuming different sample sizes (*N* = 100, 250, 500, 1000, 1500, 2000, 2500, 3000), and different heritability values (*H*^2^ = 0, 0.05, 0.1, 0.2, 0.3, 0.4). Each simulation setting is carried out 1000 times for the evaluation of power and type I error.

### Type I error evaluation and power comparison

Simulated data were used to compare our proposed tmLD method with single-marker based association analyses, including the single-marker LD mapping method (smLD) and single-marker based association test (smAT), and two-marker based haplotype analysis (haplo). The smLD was performed as described in Additional file 4. The smAT is a simple linear regression model with phenotypic trait as response variable and marker genotypes as categorical independent variable. The haplotype analysis was conducted as described in [16]; briefly, the haplotype that yields the best model fitting among those formed by two markers is used in comparison with tmLD.

**Figure 2:**
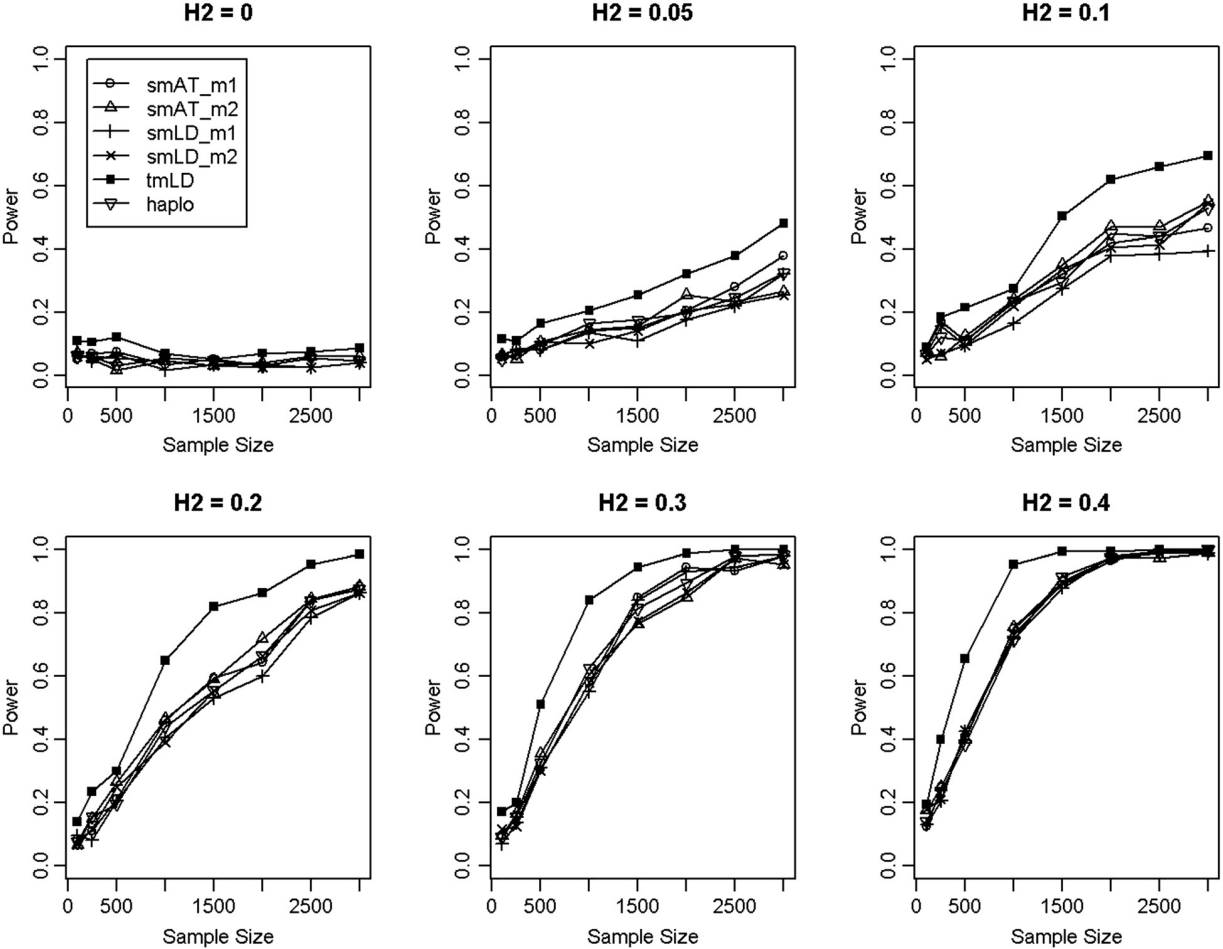
**Power comparison when QTL is in linkage disequalibria with both marker 1 and marker 2.** The power curves were constructed under different heritability (H^2^). smAT_m1 and smAT_m2 denote the single-marker association analyses for marker 1 and marker 2, respectively; smLD_m1 and smLD_m2 denote single-marker LD mapping using marker 1 and marker 2, respectively; and haplo is for the two-marker based haplotype analysis.

Under the simulation scenario 1, where the QTL is in LD phase with both markers, the results suggest that the association analysis based on two markers is significantly higher than the single- marker based and also haplotype based methods. Figure 2 shows that as the heritability increases, the power of each method increases correspondingly as expected. When *H*^2^ = 0, which suggests no QTL effects, all methods maintained the nominal type I error (0.05); when *H*^2^ ≠ 0, the two-marker association performed consistently better than others, and as expected, the power increased with the sample size.

**Figure 3:**
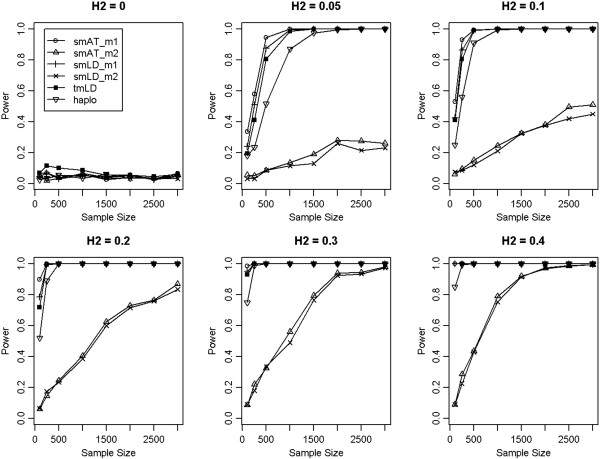
**Power comparison when QTL is at the exact position of the marker 1.** The power curves were constructed under different heritability (H^2^). The tmLD model performs almost identically with the true model even when the QTL is the marker 1. smAT_m1 and smAT_m2 denote the single-marker association analyses for marker 1 and marker 2, respectively; smLD_m1 and smLD_m2 denote single-marker LD mapping using marker 1 and marker 2, respectively; and haplo is for the two-marker based haplotype analysis.

Under the simulation scenario 2, where the QTL is set to be the marker 1, the most powerful test is the single marker association method using marker 1, and the power of the single marker association based on marker 2 is significantly lower (Figure 3). However, the tmLD analysis is almost as powerful as the optimal test, particularly when the sample size is reasonably large (N > 1000). This demonstrates that even when the QTL is indeed sampled in a genomic study, our proposed model is as good as the optimal test. These simulation results demonstrate the power advantage and robustness of our proposed method comparing with existing methods based on single marker. Its practical usage was further elucidated in a real GWAS data set.

## Real data example

Dental caries or cavities, more commonly known as tooth decay, is one of the most common chronic disorders in humans*,* affecting approximately 40% children and adolescents and 90% adults in the US. The etiology and pathogenesis of dental caries have been determined to be multifactorial, such as environmental factors related to social behaviors [17]. However, it is also apparent that some individuals are very susceptible to caries while some others are more resistant, almost irrelevant to the environmental risk factors they are exposed to, suggesting that genetic factors may play prominent roles in the caries development. Supported by evidence in both human and animal studies [18-21], the caries heritability has been estimated to be between 30-60%. The most compelling evidence come from the twin studies that the significant resemblance of dental caries lies within monozygotic but not dizygotic twin pairs [22,23]. So it is without question that in addition to environmental factors, genetic components also profoundly influence the dental caries trait. To understand the genetic mechanisms of the dental caries, a GWAS study has been conducted and the dataset has been deposited in dbGaP (Study Accession: phs000095.v2.p1). Here we will apply our proposed model to analyze this caries GWAS dataset, in which 1843 adults were genotyped with a large panel of SNPs (610,000). We carried out the analysis using the caries outcomes that have been well defined in other GWAS studies, i.e. the D1MFT index which quantifies the total permanent tooth caries with white spots.

**Figure 4:**
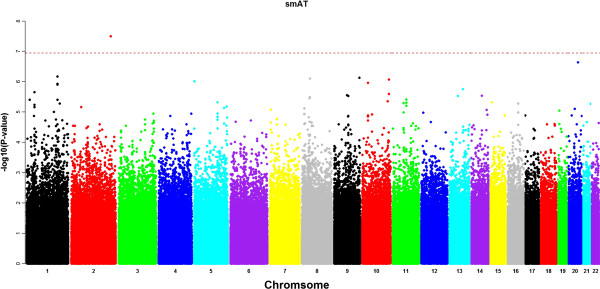
**The Manhattan plot for GWAS scanning using the single marker association analysis.** The x-axis displays the genomic coordinate of SNPs and the y-axis shows the negative base-10 logarithm of the association p-value for each SNP.

**Figure 5:**
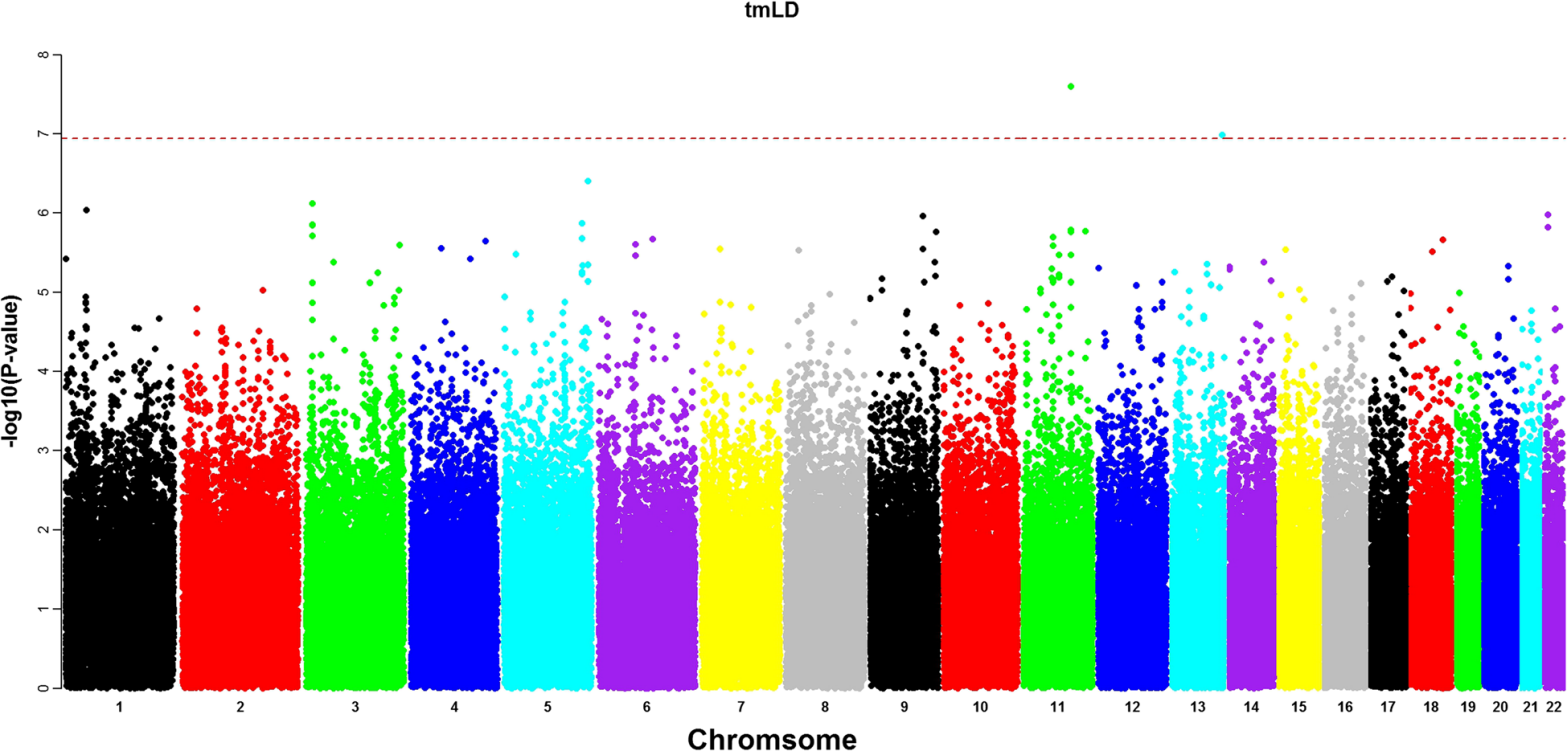
**The Manhattan plot for GWAS scanning using the two-marker LD mapping analysis.** The x-axis displays the genomic coordinate of SNPs and the y-axis shows the negative base-10 logarithm of the association p-value for each SNP.

smAT, smLD, haplo and tmLD association methods were applied to the data. After removing SNPs that do not satisfy HWE (p-values < 10^-7^) and also SNPs with minor allele frequency less than 0.1, the number of SNPs that were included in the analysis is 443,175. To compare the performance of all methods, we plotted out the association signals at each SNP locus. Figures 4 and 5 show the Manhattan plots of the -log10(p-values) from smAT and tmLD methods, respectively, and the dashed red line corresponds to the genome-wide Bonferroni threshold (1.1E-7). SNPs that passed this threshold are considered to be significant and were tabulated in Table 2. For the haplo and smLD methods, since no significant SNP was identified by these two methods, their Manhattan plots were not shown. Particularly, the tmLD model identified two significant genes, CNTN5 and COL4A2, which have been shown from other studies to be associated with dental related phenotypes in other studies [24], validating the findings of our model biologically. None of the other three methods (smAT, smLD or haplo) found these two genes. The smAT identified another significant locus. However, gene annotation shows that it is not related to any known genes, so its biological implication remains unclear.

**Table 2:** List of significant SNPs with p-value < 1.1e-7 in the Caries dataset

**SNP ID**	**Gene**	**Chr**	**Coordinate**	**Allele**	**MAF**	**P**_**smAT**_	**P**_**smLD**_	**P**_**haplo**_	**P**_**tmLD**_
rs7607421	–	2	220500564	C/T	0.390	3.2E-08^*^	2.1E-04	2.0E-06	6.9E-05
rs10790497	CNTN5	11	98539071	A/G	0.346	8.8E-01	8.2E-01	1.7E-03	**2.6E-08**^**‡**^
rs7319311	COL4A2	13	109828579	A/G	0.326	5.8E-02	2.7E-02	2.8E-02	**1.0E-07**^**‡**^

P_smAT_, P_smLD_, P_haplo_, P_tmLD_: p values for corresponding methods. ^*^Significant SNPs identified by smAT. ^**‡**^Significant SNPs identified by tmLD.

## Discussion

It is well recognized that naturally occurring variations in most complex disease traits have a genetic basis and consequently many GWAS studies have been conducted in the past few years. In analyzing these data, a phenomenon, called “missing heritability”, has been observed that the detected genetic variants can explain only a small portion of the heritability of phenotypic traits while a majority part remains mysterious [25]. Part of the reason may be attributed to the lack of power in current methods. Thus, developing novel and powerful methods to better detect significant genes has been of great interest. Currently the routine GWAS analyses seek single-marker association between SNPs and phenotype, and when a significant association is detected, it implies that there might be some SNP(s) in linkage that are causal. Note that it cannot imply the test SNP itself is causal because there is no guarantee that the truly causal SNPs would have been genotyped. Since the interpretation of a significant association relies on the linkage concept, it is sensible to directly incorporate the LD information into association models. Additionally, due to the structure of LD blocks, a causal SNP is usually in linkage with multiple neighboring SNPs, all of which carry partial information about it. So in this sense, a new model that can incorporates more genetic information of linked SNPs should draw better inferences about the causal SNP.

In this article, we proposed a novel statistical method by considering two SNPs simultaneously. Our model is built upon the general LD mapping framework, and extends the previous methods based on single-marker LD. The simulation studies demonstrated that our new methods dramatically improved the detection power of the underlying QTLs. This is intuitively reasonable since our model can capture the linkage information between SNP markers, and hence has more power to detect the particular QTL that are in LD with both markers. Furthermore, the simulation studies indicated that even when the underlying QTL is indeed genotyped and is one of the markers, the performance of the tmLD analysis is nearly identical to that of the optimal test resulting from the causal SNP, suggesting the robustness of our model.

We applied our model to a GWAS date set that aimed to understand the genetic mechanisms of the dental caries. The data set contains a large cohort of 1,843 subjects as well as a very large number of SNPs (443,175). This shows that both our proposed method and the corresponding software package in R can be well applied to a typical GWAS data set. In addition, we also observed that the association analyses based on the single-marker and the two-marker models yielded different profiles of significant SNPs. This is somewhat expected since their assumptions are different. For the tmLD method, we assume that both markers must obey HWE and have to be in LD with the casual SNP. It might be possible that some SNPs would violate these assumptions and become unsuitable to the tmLD. In this sense, the single and two-marker analyses may be complementary to each other, and therefore it might be beneficial to use both methods in analyzing a real data set.

Sometimes population structure may be a concern in a GWAS analysis if subpopulations indeed exist in the sample, as it may lead to spurious associations. Several well-known methods developed to account for population structure [26] can be incorporated into our LD mapping framework to address this issue. For instance, the principal component analysis (PCA) can be applied to correct for stratifications [27]. That is, we may first apply PCA on the genotype data and then choose the first few large principal components to be included in the Model (3) as additional covariates. With slight modifications, the computation algorithms and hypothesis testing described in the Method section can be readily applied.

In this work, we generalized the single marker LD analysis to a more general LD mapping framework using two adjacent markers. There are several ongoing works worthy of further investigation. First, the model can be easily extended to other types of phenotypic data, such as case–control binary and count data. Second, currently the two adjacent markers were used for the analysis; however, it is possible that another two markers in the same LD block might have better power, so it would be very interesting to determine how to choose the best SNP pair. Third, typically, one LD block may contain several SNPs, and if there exists one causal SNP within the LD block, it would be very interesting to see if we can summarize all SNPs in one LD block to make even better inference about the unobserved QTL.

## Conclusions

The proposed tmLD model is a novel mapping method that can simultaneously consider two linked SNPs in a natural population. Through the extensive simulation studies, the tmLD method demonstrates better power than single-marker mapping strategies traditionally used in GWAS association analysis. The practical usage of the tmLD method was also shown in the analysis of a large-scale dental GWAS dataset. Hence, we recommend the usage of this improved method over the traditional single-marker association analysis.

### Software availability

http://www.ams.sunysb.edu/~songwu/software.html.

## Abbreviations

LD: Linkage disequilibrium; SNP: Single-nucleotide polymorphism; QTL: Quantitative trait loci; GWAS: Genome-wise association study; smAT: Single-marker association test; smLD: Single-marker linkage disequilibrium method; tmLD: Two-marker linkage disequilibrium method; haplo: Two-marker based haplotype analysis; MAF: Minor allele frequency; HWE: Hardy-Weinberg equilibrium.

## Competing interests

No competing interests exist for any author.

## Authors' contributions

JY conceived of the study, performed the statistical analysis and drafted the manuscript. WZ conceived of the study and drafted the manuscript. JC and QZ performed the statistical analysis and drafted the manuscript. SW conceived of the study, performed the statistical analysis, drafted the manuscript and developed the R package. All authors have read and approved the final manuscript.

## Supplementary Material

Additional file 1 Representation of three-loci haplotypes with four LD parameters. Click here for file

Additional file 2 **Derivation of how *D***_**123 **_**may change with time.** Click here for file

Additional file 3 Derivation of the EM algorithm used to find MLEs for a mixture model. Click here for file

Additional file 4 Single-marker based LD mapping. Click here for file
