# Supplementary material for: Genome-wide Two-marker linkage disequilibrium mapping of quantitative trait loci
Source: BMC Genet. 2014 Feb 8;15:20. doi: 10.1186/1471-2156-15-20 (PMC4015628; doi:10.1186/1471-2156-15-20)
Supplement: Additional file 1 — Representation of three-loci haplotypes with four LD parameters. [file 1471-2156-15-20-S1.doc]

**Additional file 1: Representation of three-loci haplotypes with four LD parameters**

The three SNPs
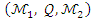
form 8 possible haplotypes:
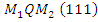
,
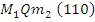
,
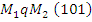
,
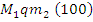
,
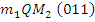
,
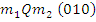
,
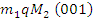
,
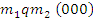
. To describe the linkage disequilibrium among them, a set of trigenic disequilibria parameters can be introduced to describe their frequencies as follows:


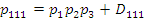


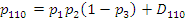


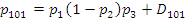


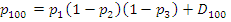


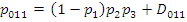


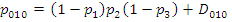


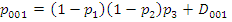


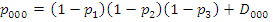


However, with the constraints that


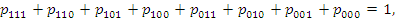


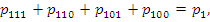


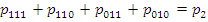
,


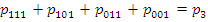
.

Half of these
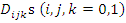
 are actually redundant. With some simple algebra, it is straightforward to show that the haplotypes’ frequencies can be rewritten using four trigenic disequilibria parameters
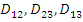
 and
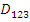
 as follows:


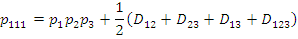


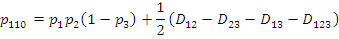


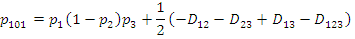


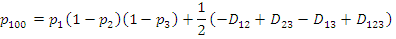


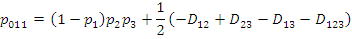


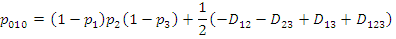


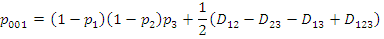


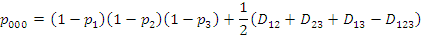


Where
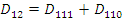
,
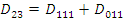
,
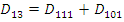
 and
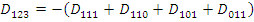
.

In a more compact form, The above equations can be rewritten as:


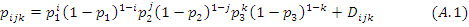


and
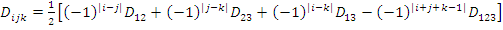


where
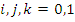
.

Notice that the trigenic model is fully compatible with the lower-level digenic model. That is, the LD parameters of
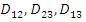
have exactly the same meaning as those in digenic disequilibria models for loci at positions 1/2, 2/3 and 1/3, respectively. For example, adding
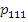
 and
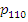
,
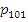
 and
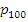
,
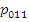
 and
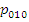
, and
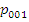
 and
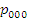
 respectively yield the haplotype frequencies for the first two loci:


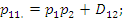


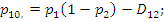


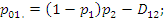


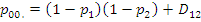


where,
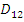
 fully describes the linkage disequilibria between loci 1 and 2 and similar arguments can be made for
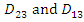
. Based on the theory for the digenic LD model, under the Hardy-Weinberg Equilibrium that
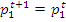
,
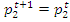
 and
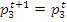
, the
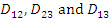
 will decrease in generations at a slow rate as shown in Equation (1) in the main text.
