# Supplementary material for: Genome-wide Two-marker linkage disequilibrium mapping of quantitative trait loci
Source: BMC Genet. 2014 Feb 8;15:20. doi: 10.1186/1471-2156-15-20 (PMC4015628; doi:10.1186/1471-2156-15-20)
Supplement: Additional file 2 — Derivation of how D 123 may change with time. [file 1471-2156-15-20-S2.doc]

**Additional file 2: Derivation of how**
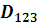
 **may change with time.**

suppose the three linked SNPs,
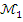
,
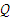
 and
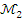
, in a tandem order at loci 1, 2 and 3, and the recombination rates between
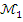
 and
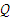
, between
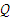
 and
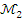
, and between
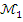
 and
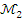
 are
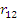
,
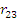
 and
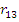
. In a typical SNP array, their physical distances and recombination rates are usually very small. For example, the average distance between two SNPs in Affymetrix SNP6.0 array is about 3kb, corresponding to an approximate recombination rate of 0.00003. Based on the Kosambi's mapping function, in a very small region, the recombination rate between
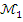
 and
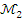
 can be expressed as the sum of the recombination rates in the two subregions, *i.e.*,
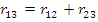
, which basically states that in such a small region, the probability of having two recombination events simultaneously in a small region is unlikely. Let the frequencies of the eight gametes generated from the three SNPs at generation
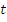
 be expressed as
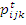
, where
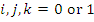
. For a particular gamete, say
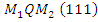
, it has a proportion of
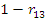
 in the next generation
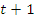
 produced without recombination and the frequency is
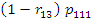
. This gamete can also be generated as a recombinant form from genotypes formed by gametes containing allele
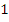
 of
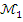
 and allele
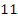
 of
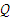
 and
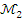
, or by gametes containing allele
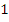
 of
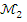
 and allele
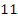
 of
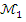
and
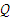
. The frequency with which
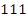
 arises from recombination events is
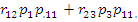
. Therefore the frequency of
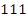
 in the generation
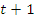
 is


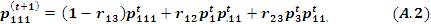


If we assume all loci satisfy the Hardy-Weinberg equilibrium (HWE), the allele frequencies are constant from generation to generation. Expanding both sides of the equation (A.2) using equation (A.1) yields


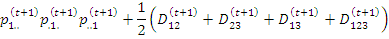


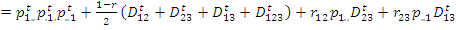
 .

Then, replacing
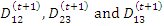
 in the above equation using equation (1) yields


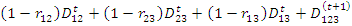


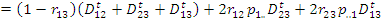
 .

Since
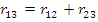
, canceling terms on both sides yields,


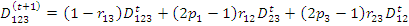
.

Because
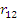
,
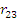
 and
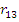
 are very small, at the level of
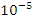
, the last two terms on the right side of equation, i.e.,
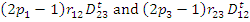
, are negligible. Therefore, we can have


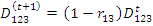
.

So, approximately, the
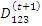
 decreases with generations at a rate of
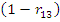
 and changes very slowly with time.
