# Supplementary material for: Genome-wide Two-marker linkage disequilibrium mapping of quantitative trait loci
Source: BMC Genet. 2014 Feb 8;15:20. doi: 10.1186/1471-2156-15-20 (PMC4015628; doi:10.1186/1471-2156-15-20)
Supplement: Additional file 3 — Derivation of the EM algorithm used to find MLEs for a mixture model. [file 1471-2156-15-20-S3.doc]

**Additional file 3: Derivation of the EM algorithm used to find MLEs for a mixture model.**

We need to maximize the log-likelihood in the form below:


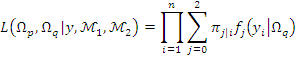


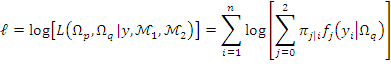


Let us augment the data with a group of indicator variables
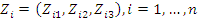
, where


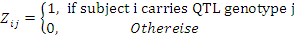
 and
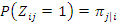
.

So,
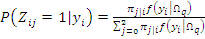


Then the joint (complete) likelihood of
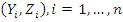
 is therefore


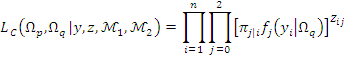


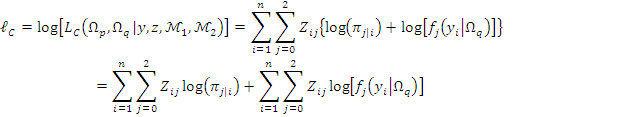


In the E-step,


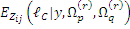


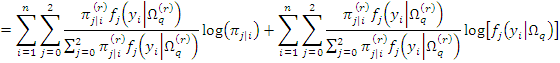


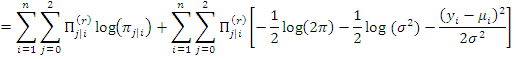


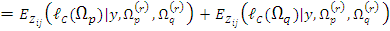


So
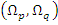
 in
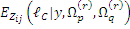
 are separated into two terms, which can be maximized individually.

Hence, In the M-step,

M1. to maximize


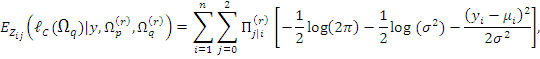


it is easy to verify that:


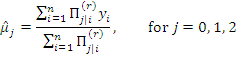


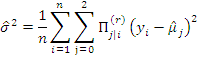


M2. to maximize the
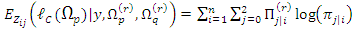
, which is actually the kernel of a multinomial distribution, we can apply another cycle of EM algorithm to obtain the MLE of
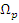
. First, define the expected counts for each genotype as,


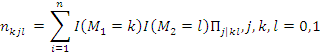


In the E-step, let


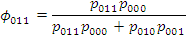


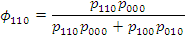


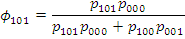


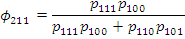


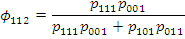


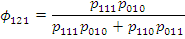


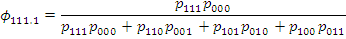


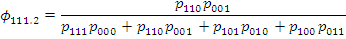


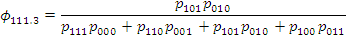


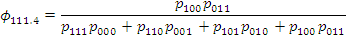


In the M-step, the haplotype frequencies can be estimated by:


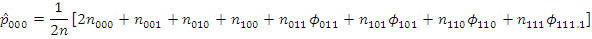


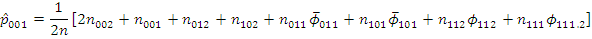


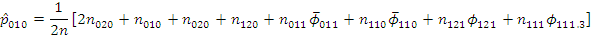


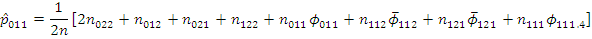


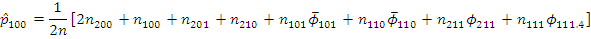


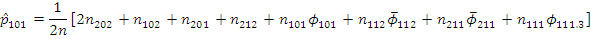


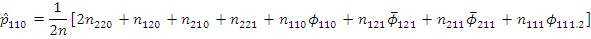


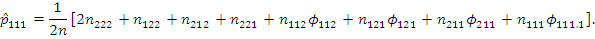


With the MLEs of the above haplotype frequencies, the MLEs of the corresponding allele frequencies and linkage disequilibria among the markers and QTL can be obtained.
