# Supplementary material for: Genome-wide Two-marker linkage disequilibrium mapping of quantitative trait loci
Source: BMC Genet. 2014 Feb 8;15:20. doi: 10.1186/1471-2156-15-20 (PMC4015628; doi:10.1186/1471-2156-15-20)
Supplement: Additional file 4 — Single-marker based LD mapping. [file 1471-2156-15-20-S4.doc]

**Additional file 4: Single-marker based LD mapping**

Suppose there is a dichotomous QTL of alleles
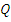
 and
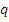
 that is causal, but unobserved, to a phenotype of interest
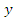
. For illustration, let’s assume
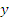
 is a continuous variable. The allele frequencies of
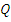
 and
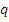
 are expressed as
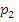
and
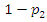
, respectively. Three genotypes,
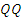
,
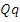
 and
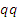
, can be formed for this QTL. Suppose this QTL is genetically associated with a genotyped SNP marker
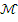
 of two alleles
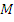
 and
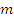
, with corresponding frequencies of
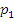
 and
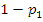
. The SNP marker can also have three genotypes:
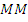
,
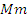
 and
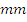
. The marker and QTL form four haplotypes:
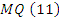
,
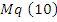
,
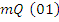
 and
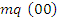
, with corresponding frequencies expressed as
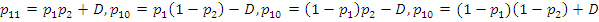
, where
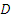
 is the LD coefficient between the marker and QTL. Statistically, the phenotypic value for subject
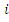
,
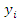
, at a putative QTL can be expressed in a mixture model:


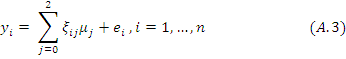


where
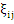
 is an indicator variable defined as 1 if subject
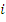
 has a QTL genotype
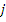
 (
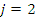
 for
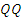
,
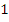
 for
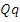
 and
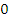
 for
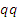
) and 0 otherwise,
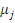
 is the expected phonotypic value for QTL genotype j, and
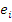
 is the error term reflecting the polygenic effects of other unlinked genes and the environmental effect, which can be assumed to follow
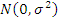
 if
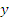
 is continuous. The conditional probability of subject
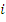
 with its given marker carrying QTL genotype
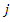
,
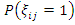
, can be calculated from the joint probability distribution of marker and QTL (Table A.1).

Then, based on Model
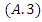
, a hypothesis testing can be constructed as below to test if a QTL is associated with the given marker:


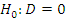
 *vs.*
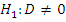
.

A likelihood ratio test statistics that asymptotically follow a chi-squared distribution with one degree of freedom can be used to assess the test significance. Specifically, the detection of a significant non-zero
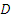
 value implies that a QTL may be genetically linked with
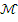
.

Table A.1 : The joint genotype frequencies of one marker and one QTL

|  | 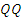 | 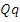 | 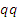 |
| --- | --- | --- | --- |
| 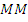 | 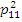 | 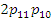 | 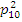 |
| 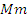 | 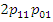 | 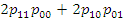 | 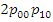 |
| 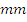 | 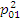 | 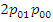 | 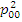 |
